# Experimental screening and structure-informed engineering of peptide-fused bacteriophage lysins with enhanced activity against Gram-negative bacteria

**DOI:** 10.3389/fphar.2026.1918219

**Published:** 2026-09-21

**Authors:** Ali Murtaza, Fangfang Yao, Aqib Saeed, Xiaohong Li, Nelson Odiwuor, Xinjing Du, Changchang Li, Junhua Li, Hongping Wei

**Affiliations:** 1 WHP Innovation Lab, State Key Laboratory of Virology and Biosafety, Wuhan Institute of Virology, Chinese Academy of Sciences, Wuhan, China; 2 University of Chinese Academy of Sciences, Beijing, China; 3 Stomatological Hospital of Xiamen Medical College, Xiamen Key Laboratory of Stomatological Disease Diagnosis and Treatment, Xiamen, China

**Keywords:** *Acinetobacter baumannii*, AlphaFold-based structure prediction, lysin, positive charge residues, α-helical antimicrobial peptides

## Abstract

The increasing prevalence of antibiotic-resistant bacteria necessitates the development of novel antimicrobial strategies. Bacteriophage-derived lysins are promising options, but lysins against Gram-negative bacteria lose their activity under physiological conditions. Peptide fusion strategies have been used to address this problem with limited success. In this study, structure prediction and charge analysis were used to guide the selection of suitable peptides for engineering lysins against Gram-negative bacteria. Seven variants of LysPd138 were constructed by fusing it with different peptides. Their activities were tested in 150 mM NaCl and human and mouse serum. The variants (Syn138, Pad138, and CecA138) showing enhanced activity in 150 mM NaCl solution and in sera were found to be fused with peptides, with most of their residues forming α-helical structures, as suggested by computational structure analysis using AlphaFold and PSIPRED. In contrast, the inactive variants were fused to peptides predicted to adopt non-helical structures and possess a relatively small number of positively charged residues. This observation suggested that the presence of more α-helical structures within a positively charged fused peptide may be an important feature for designing effective lysin fusions under physiological conditions. This was further examined by fusing two selected peptides with another lysin, LysPd149. These findings suggest that protein structure prediction and charge analysis could provide a more efficient way to rationally select or design peptides to be fused with lysins against Gram-negative bacteria before protein expression, saving time and costs associated with the current trial-and-error fusion strategies.

## 1 Introduction

Antimicrobial resistance represents a serious, growing, and emerging global health threat in the 21st century. From 1990 to 2021, an estimated one million deaths were reported each year due to these drug-resistant pathogens. A report published in The Lancet warns that, without effective interventions, antibiotic-resistant infections could lead to an estimated 39 million deaths by 2050 (Naghavi et al., 2024).

Lysins are bacteriolytic enzymes encoded by bacteriophages that may offer a solution to the antimicrobial resistance challenge. These enzymes are naturally produced during the terminal phase of the bacteriophage lytic cycle and play a key role in degrading peptidoglycan (Young et al., 2000; De Smet et al., 2017; Abdelrahman et al., 2021). Lysins can be expressed and purified in the laboratory using recombinant methods. They exhibit strong antibacterial and anti-biofilm efficacy when applied externally (Lendel et al., 2024; El-Telbany et al., 2026; Xiang et al., 2024). To date, lysins are reported to be non-toxic against eukaryotic cells and highly potent in combating resistance mechanisms (Wang et al., 2023; Wang et al., 2025; Moghadam et al., 2025). Numerous studies have identified lysins against Gram-positive bacterial pathogens, which have the potential to be implemented in therapeutic strategies and the food industry, such as for managing *Staphylococcus aureus* infections (Schmelcher and Loessner, 2016; Ramos-Vivas et al., 2021; Bae et al., 2019).

Unlike Gram-positive bacteria, the peptidoglycan in Gram-negative bacteria is enclosed by an outer membrane enriched with lipopolysaccharides. The outer membrane in Gram-negative bacteria reduces the bactericidal activity of lysins by blocking their entry to peptidoglycan (Gontijo et al., 2021). Although several lysins have been identified with strong intrinsic antibacterial activity against Gram-negative pathogens under low-tonicity conditions (Khan et al., 2021; Taj et al., 2025; Yao et al., 2025), one major problem of these lysins is that they will lose bactericidal activity in high concentrations of sodium chloride and biological fluids such as human serum, limiting their applications (Yao et al., 2025). Protein engineering has been explored to enhance the activity of lysins against Gram-negative bacteria (Wojciechowska, 2025; Zhang et al., 2026). For example, engineering lysins with positively charged peptides have been used to screen variants with enhanced activity (Sui et al., 2023; Yao et al., 2025). Similarly, it has been demonstrated that enriching the C-terminal helix with surface-positive charge substantially enhances the antibacterial efficacy of lysin by boosting its contact with the outer membrane of Gram-negative bacteria (Kim J. et al., 2025). However, the current trial-and-error strategy needs a lot of screening experiments to find suitable peptides, which is costly and labour-intensive.

In recent years, breakthroughs in artificial intelligence (AI) have greatly improved the ability of using computer to predict protein structures and interactions between proteins and other molecules (Jumper et al., 2020), therefore, providing a new opportunity to understand the changes associated with activity after fusing different peptides with a lysin. Such knowledge may provide rational design or choice of suitable peptides to engineer a lysin with better activity in biological fluids.

In the current study, two lysins, LysPd138 (Yao et al., 2025) and LysPd149 (Yao et al., 2026), identified with intrinsic antibacterial activity against *Acinetobacter baumannii* by our lab, were chosen. First, LysPd138 was fused with seven different positively charged peptides. Varied activities were found in solutions with different NaCl concentrations, as well as in mouse and human serum. Protein structure prediction and charge analysis were performed to investigate the structural features that may contribute to the varied activities. AlphaFold and PSIPRED-based structure prediction suggested that strong bactericidal activity may be related to the α-helical structure of the fused peptides. This observation was further supported by engineering LysPd149 with a peptide predicted to adopt an α-helical structure and enriched in positively charged residues.

## 2 Materials and methods

### 2.1 Bacterial strains, reagents, and growth conditions

*A. baumannii* WHG40137 was obtained from Tongji Hospital (Wuhan, China). For cloning and protein purification, *E. coli* (*Escherichia coli*) BL21 was used. The human serum and plasma were purchased commercially, and the mouse serum was collected from BALB/c mice that were used in an experiment in our lab. All bacterial strains were cultured in Lysogeny broth (LB) at 37 °C with shaking at 200 rpm. Kanamycin was added at a concentration of 50 μg/mL when needed.

### 2.2 Plasmid construction

An overview of all the peptides and engineered lysins produced in this study is provided in Table 1. LysPd149 (Yao et al., 2026) and LysPd138 (Yao et al., 2025) were previously characterized in our lab. Engineered variants were constructed by fusing a peptide to the N-terminus of the lysin using an AGAGAG linker (Figure 1). The full coding sequence of engineered variants of LysPd138 and LysPd149 was assembled using overlap extension PCR as described previously (Yao et al., 2025). The engineered lysins comprise three modules arranged from the N-terminus to the C-terminus: a peptide, a flexible (AG)_3_ linker, and the LysPd138 or LysPd149 lysin. Recombination with the pET28a vector was carried out using the ClonExpress Ultra One-Step (C116-02, Vazyme, Nanjing, China) (Zeng et al., 2024), yielding recombinant pET28a-engineered lysin plasmids. PCR and DNA sequencing confirmed correct clones, and verified constructs were used for subsequent experiments.

**TABLE 1:** Overview of engineered lysins.

Name of engineered variant	Lysin	Linker	Peptide
CecA138 CecA149	LysPd138 LysPd149	AGAGAG	CecA (Wang et al., 2019)
Syn138	LysPd138	AGAGAG	SynSaf-P56 (Fields et al., 2020)
Pad138	LysPd138	AGAGAG	PaDBS1R6 (Rezende et al., 2024)
Buf138 Buf149	LysPd138 LysPd149	AGAGAG	Buforin-2 (8–21) (Park et al., 2000)
G8138	LysPd138	AGAGAG	G8 (Lyu et al., 2023)
E7K138	LysPd138	AGAGAG	E7K_I9K_IsCT (Tripathi et al., 2015)
DP7138	LysPd138	AGAGAG	DP7 (Jiang et al., 2022)

**FIGURE 1:**
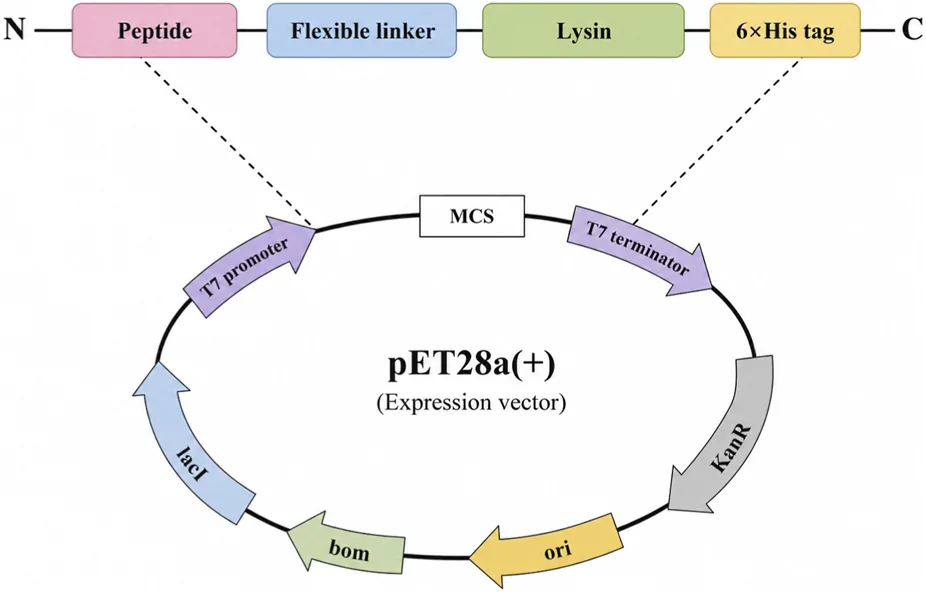
General schematic representation of engineered variants.

### 2.3 Protein expression and purification

The recombinant proteins were overexpressed as previously described, with minor modifications (Li et al., 2024). Briefly, to express the targeted lysins, *E. coli* (BL21) cultures carrying either pET28a-engineered lysins, pET28a-LysPd138 or pET28a-LysPd149, were induced with 0.2 mM IPTG at 16 °C for 16 h. Next, the cells were harvested by centrifugation and suspended in lysis buffer (Tris-HCl). Following cell lysis using a high-pressure homogenizer, the unbroken cells were centrifuged. Subsequently, the supernatant fraction was filtered through a 0.22-µm pore-size filter and applied to a nickel nitrilotriacetic acid affinity column (GE Healthcare). The C-terminally His_6_-tagged proteins were purified by washing the affinity column with 20 mM imidazole in 20 mM Tris-HCl (pH 8.0) containing 0.5 M NaCl, followed by elution with 250 mM imidazole in the same buffer. The purified proteins were then dialyzed overnight against 10 mM HEPES (pH 7.4) and stored at 4 °C until further use. Details of prepared buffers are provided in the Supplementary Material. Protein size and purity were analysed using SDS-PAGE. Protein concentration was determined using a NanoDrop instrument (ThermoFisher Scientific, NanoDrop one) or Pierce BCA Protein Assay Kit (Thermo Scientific, Waltham, MA, USA). The protein coefficient of extinction was calculated using an online tool (https://www.novoprolabs.com/tools/protein-extinction-coefficient-calculation).

### 2.4 *In vitro* antibacterial assays under different conditions

The bactericidal activity of each lysin was evaluated against *Acinetobacter baumannii* WHG40137 under physiological conditions, including high NaCl concentration and human and mouse serum, using the CFU reduction assay. Exponentially growing bacteria were harvested, washed by centrifugation, and resuspended in 10 mM HEPES buffer (pH 7.4) to an OD600 of 0.4. All antibacterial assays were performed in a final reaction volume of 200 µL. Unless otherwise stated, bacteria were first mixed with NaCl or serum, followed by the addition of the lysin solution. Untreated controls received an equivalent volume of lysin-storage buffer (10 mM HEPES) instead of lysin. Detailed reaction compositions are provided in the Supplementary Material.

Briefly, for the NaCl experiments, the bacterial suspension prepared as described above was diluted 100-fold in 10 mM HEPES buffer before being used in the antibacterial assay. Subsequently, 100 µL of the diluted bacterial suspension was mixed with either HEPES buffer or NaCl solution to achieve final NaCl concentrations of 0 or 150 mM, respectively, followed by the addition of lysin to a final concentration of 100 μg/mL. To evaluate the concentration-dependent bactericidal activity of the lysins, 100 µL of the diluted bacterial suspension was mixed with NaCl solution to achieve a final NaCl concentration of 150 mM and treated with lysin at final concentrations of 100, 85, 70, and 50 μg/mL, while maintaining a total reaction volume of 200 µL.

For the human serum assays, the prepared bacterial suspension was directly diluted 100-fold in human serum. Subsequently, for the 40% serum assay, 82 µL of the bacterial suspension prepared in human serum was mixed with HEPES buffer and lysin to adjust the final reaction volume to 200 µL. For the 80% serum assay, 162 µL of the bacterial suspension prepared in human serum was combined with lysin to achieve the desired final serum concentration. The final lysin concentration in both assays was maintained at 100 μg/mL.

For the mouse serum assays, the prepared bacterial suspension was diluted 100-fold in 10 mM HEPES buffer. Subsequently, 100 µL of the diluted bacterial suspension was mixed with mouse serum, HEPES buffer, and lysin to achieve final mouse serum concentrations of 5% or 10% (v/v), a final lysin concentration of 100 μg/mL, and a total reaction volume of 200 µL.

All reaction mixtures were incubated at 37 °C for 1 h. After incubation, samples were serially diluted in a 10-fold manner in 1× PBS, and 10 µL of the diluted samples were plated on LB agar plates for overnight incubation at 37 °C. Colonies were counted after incubation, and bacterial survival was expressed as CFU/mL. Experiments were repeated at least three times.

### 2.5 Human plasma stability assay

CecA149 and the CecA were diluted to an initial concentration of 7.6 µM in 50% (v/v) non-heat-inactivated human plasma and incubated at 37 °C for 0, 2, 3, and 5 h. As a control, 10 mM HEPES buffer was incubated under the same conditions in 50% human plasma to exclude any effect of the buffer. At each time point, 100 µL of the plasma-incubated CecA149 or CecA solution was mixed with 100 µL of exponentially growing *A. baumannii* WHG40137 prepared in 10 mM HEPES buffer as described above, resulting in final CecA149 or CecA concentrations of 3.8 µM and a final human plasma concentration of 25% (v/v). As a control, HEPES buffer incubated with plasma was combined with the same volume of bacteria. Following incubation at 37 °C for 1 h, the reaction mixtures were serially diluted, and incubated overnight at 37 °C for CFU enumeration as described above. Each experiment was performed with two independent biological replicates, each with three technical replicates.

### 2.6 Outer membrane permeabilization assay

The experiment was conducted as previously described with minor modifications (Li Q. et al., 2021; Gerstmans et al., 2023). Briefly, bacterial outer membrane integrity was assessed by performing the 1-N-phenylnaphthylamine (NPN) uptake assay. Exponentially growing *A. baumannii* WHG40137 culture was harvested and washed through centrifugation. Next, the harvested cells were resuspended to an OD600 of 0.25 in a 10 mM HEPES buffer, pH 7.4, either in the presence or absence of 300 mM NaCl. The Protein dilutions and working solution of NPN were prepared in the same buffer without NaCl. The reaction solution was prepared in a 96-well white plate (Greiner bio-one, REF:655083), consisting of 100 µL of bacterial suspension, 50 µL of NPN (final concentration of 30 µM), and 50 µL of each lysin (final concentration of 100 μg/mL). The fluorescence intensity was measured after 30 min of incubation at room temperature at an excitation and emission range of 350 and 420 nm, respectively. Two independent experiments were performed, each with three technical replicates. The NPN uptake factor was calculated using the following formula.

$$\frac{\left(Treated-Blank\right)}{\left(Negative\;control-Blank\right)}$$

Treated = fluorescence of the bacteria treated with proteins, Negative control = fluorescence of the bacteria treated with HEPES buffer, Blank = fluorescence of the HEPES buffer and NPN only.

### 2.7 Protein structure prediction, charge calculation and statistical analysis

AlphaFold is a widely used tool for protein structure prediction (Jumper et al., 2020). The three-dimensional structures of the engineered lysins were predicted using ColabFold (Kim G. et al., 2025), a local implementation of AlphaFold2. Predictions were run with the parameters provided in the Supplementary Material to ensure reproducibility. Five models were generated for each sequence, and the rank 1 model was selected for further analysis. The predicted structures were visualized using PyMOL (version 4.6.0). The secondary structures of the fused peptides were predicted using PSIPRED (http://bioinf.cs.ucl.ac.uk/psipred/). The net charge of all the peptides was predicted using an online tool (https://www.calculatorultra.com/zh/tool/protein-charge-calculator.html). Data were analysed using GraphPad Prism version 10.4.0, using the Kruskal–Wallis test, followed by Dunn’s multiple comparisons test, unless otherwise indicated individually. All data are presented as the mean ± SD, and differences were considered statistically significant when P < 0.05.

## 3 Results

### 3.1 Construction and initial characterization of LysPd138 variants

Positively charged residues within a peptide were found useful for improving the activity of lysins (Sui et al., 2023). Therefore, peptides with a high number of positively charged residues and those with a low number of positively charged residues were selected (Supplementary Table S1). These peptides were fused to the N-terminus of LyPd138 to enhance its antimicrobial activity under physiological conditions. After expression and purification, SDS-PAGE analysis showed that LyPd138 and its engineered variants were successfully obtained and their concentrations were measured using a NanoDrop instrument (Thermo Fisher Scientific, NanoDrop One) and the Pierce BCA Protein Assay Kit (Thermo Scientific, Waltham, MA, United States) (Supplementary Figure S1). As the concentrations measured by these two methods were similar, the concentration determined by NanoDrop was used in subsequent experiments.

Initially, the bactericidal activity of LysPd138 and its engineered variants was evaluated at 100 μg/mL against *A. baumannii* WHG40137 in 10 mM HEPES buffer without 150 mM NaCl. In the absence of NaCl, all engineered variants reduced *A. baumannii* WHG40137 to below detectable levels (Supplementary Figure S1). Notably, *A. baumanni*i WHG40137 is a drug-resistant strain, and its antibiotic resistance profile has been characterized previously (Li C. et al., 2021).

### 3.2 Bactericidal activity of LysPd138 engineered variants under high-NaCl conditions and structural analysis

Next, we evaluated the antibacterial activity of LysPd138 and its engineered variants against *A. baumannii* WHG40137 at 100 μg/mL in 10 mM HEPES buffer (pH 7.4) containing 150 mM NaCl. In the presence of 150 mM NaCl, LysPd138 and its other engineered variants significantly lost their bactericidal effects (Figure 2A), whereas Syn138, Pad138, and CecA138 eradicated the bacteria to below the detection limit (Figure 2C). In addition, we selected two active engineered variants, CecA138 and Syn138, along with the native lysin LysPd138, to evaluate the effect of protein concentration on bactericidal activity. The activity was tested against *A. baumannii* WHG40137 at different concentrations (100, 85, 70, and 50 μg/mL) in the presence of 150 mM NaCl (Figure 2D). Results revealed that the activity is dose-dependent for CecA138, while LysPd138 did not show activity at any tested concentration. CecA138 significantly reduced the bacterial count in a dose-dependent manner. Syn138 reduced the bacterial count by 4-log at all tested concentrations, indicating its strong bactericidal effect even at low concentrations.

**FIGURE 2:**
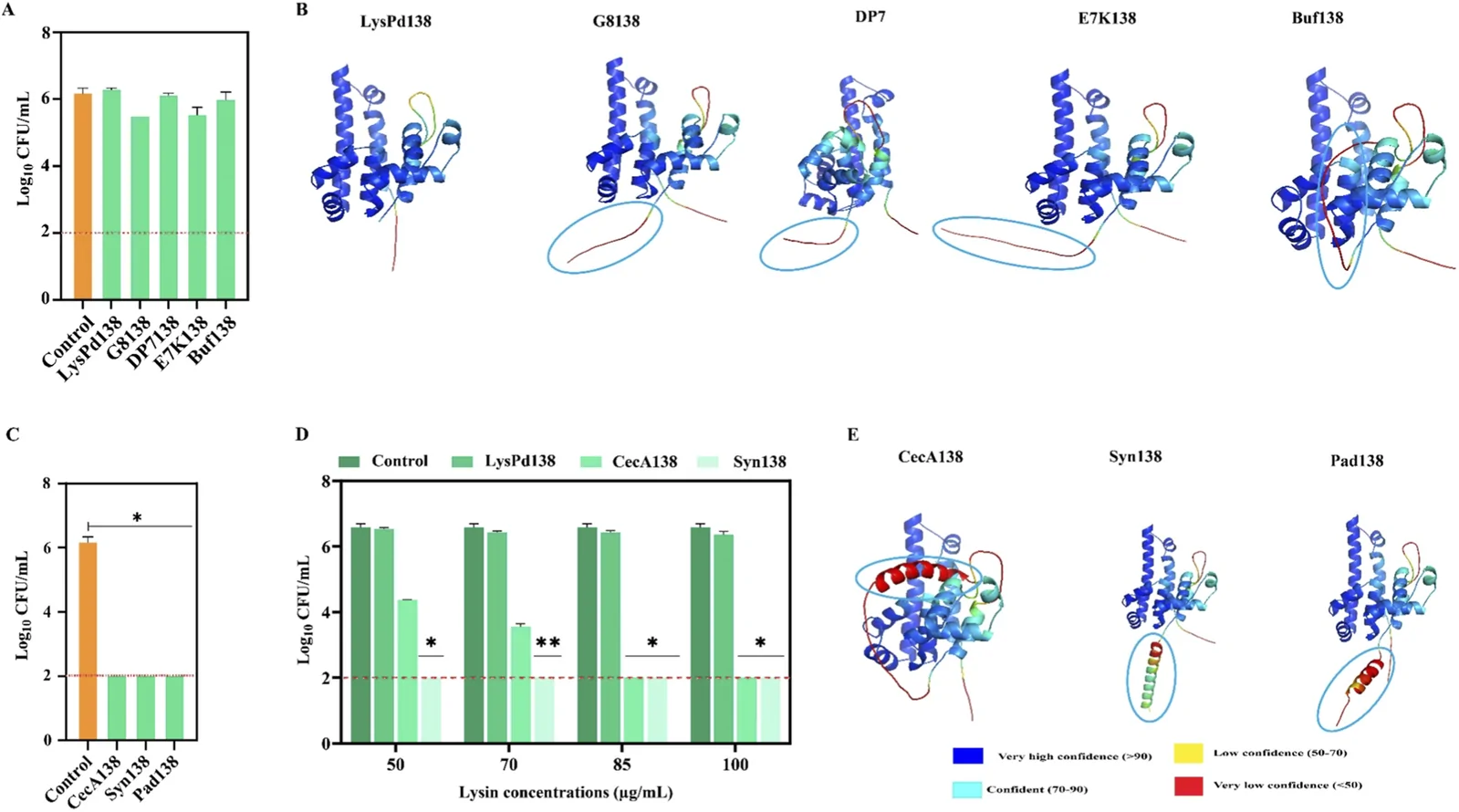
Effect of 150 mM NaCl on the bactericidal activity of LysPd138 and its engineered variants. *Acinetobacter baumannii* WHG40137 was treated with 100 μg/mL of each lysin, unless otherwise indicated, in the presence of 150 mM NaCl for 1 h at 37 °C. **(A)** Engineered variants showing weak activity in presence of 150 mM NaCl. **(B)** AlphaFold-based predicted structures of engineered variants which are showing weak activity in 150 mM NaCl. **(C)** Engineered variants showing strong activity in presence of 150 mM NaCl. **(D)** *Acinetobacter baumannii* WHG40137 was treated with different concentrations of lysin in presence of 150 mM NaCl. **(E)** AlphaFold-based predicted structures of engineered variants which are active in 150 mM NaCl. The region enclosed in the circle indicates the fused peptide. The dashed line indicates the limit of detection. Data are presented as mean ± SD of three technical replicates from one representative experiment. Only statistically significant differences between treated groups and the untreated control are indicated by asterisks. Statistical analysis was performed using the Kruskal–Wallis test followed by Dunn’s multiple comparisons test based on the three technical replicates. *p < 0.05, **p < 0.01.

To understand factors involved in the interactions of lysin with bacteria that may lead to varied activities after fusion with different peptides, computer tools were used to compare the changes after peptide fusion. The structures of the lysins were predicted using Alphafold 2. Structural analysis of these variants revealed that G8, DP7, E7K_I9K_IsCT, and Buforin-2 (8–21) adopt a loop-like structure upon fusion with LysPd138, resulting in G8138, DP7138, E7K138, and Buf138 engineered variants, respectively (Figure 2B). Conversely, many of the amino acid residues in SynSaf-P56, PaDBS1R6, and CecA adopt α-helical structure upon fusion with LysPd138, resulting in Syn138, Pad138, and CecA138 engineered variants, respectively (Figure 2E). Notably, the pLDDT confidence scores for these fused peptide regions were mostly very low (e.g., <50). Only the fused peptide in Syn138 has a pLDDT confidence of more than 70 (Supplementary Figure S2).

To independently assess the secondary structure propensity of the fused peptides, we analyzed their sequences using PSIPRED, a method specialized in predicting secondary structure from sequence. The structure analysis revealed that SynSaf-P56, PaDBS1R6, and CecA have high α-helical content, whereas G8, DP7, E7K_I9K_IsCT, and Buforin-2 (8–21) were predicted to have little to no helical structure and instead showed high coil propensity (Supplementary Figure S3).

Furthermore, it has been observed that engineered variants with enhanced bactericidal activity under physiological conditions contain a fused peptide with relatively high positive charges compared with peptides fused with less active variants (Supplementary Table S1). The more positively charged fused peptides are likely to interact more strongly with the negatively charged outer membrane of bacteria, thereby enhancing the antibacterial and permeability activity of lysin even in the presence of high NaCl concentrations.

### 3.3 Effect of serum on bactericidal activity of LysPd138 engineered variants

Next, the bactericidal effect of those engineered variants which showed enhanced bactericidal activity in presence of 150 mM NaCl was evaluated in 40% and 80% human serum at 100 μg/mL (Figure 3A). Pad138 reduced the bacterial count to below the detection limit in 40% human serum but was less active in 80% human serum, reducing the bacterial count by only ∼0.4 log compared with the control group. In contrast, Syn138 and CecA138 eliminated the bacteria to below the detection limit at both tested human serum concentrations (Figure 3B). Additionally, the bactericidal activity of LysPd138, Syn138, and CecA138 was evaluated in different concentrations of mouse serum against *Acinetobacter baumannii* WHG40137. Results revealed that Syn138 and CecA138 could eliminate *A. baumannii* WHG40137 to below the detection limit in 5% serum; however, 10% serum significantly inhibited the bactericidal activity of both engineered lysins (Figure 3C). LysPd138 did not exhibit bactericidal activity at either tested concentration. These findings indicate that mouse serum inhibits the bactericidal activity of the engineered lysins to a greater extent than human serum.

**FIGURE 3:**
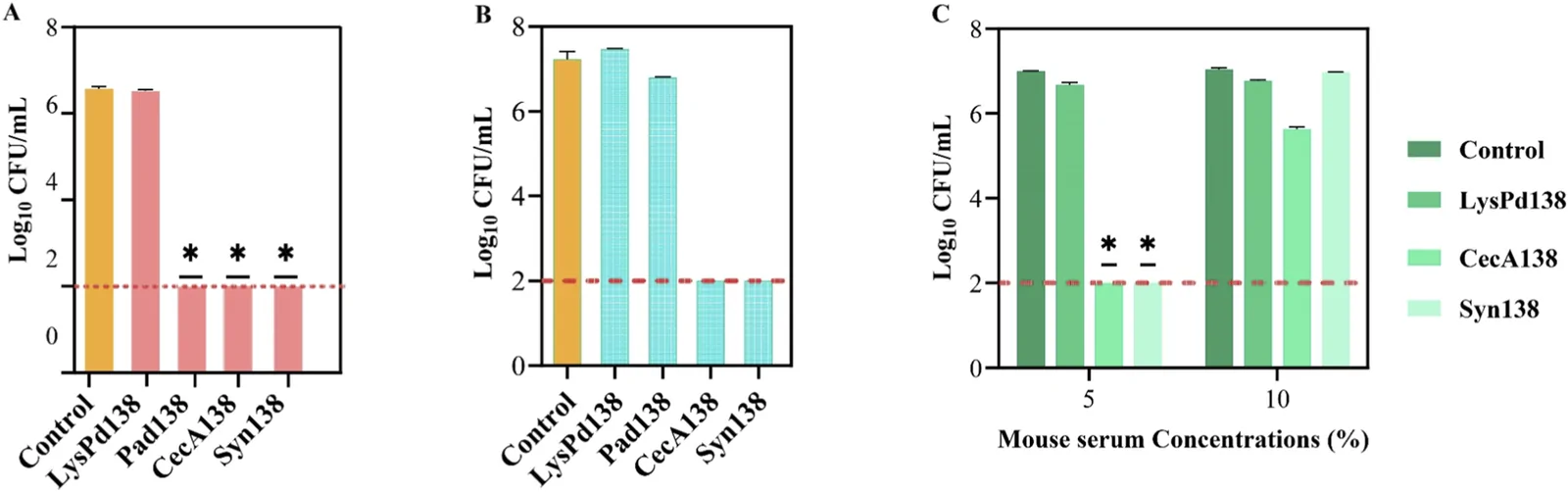
Bactericidal activity of LysPd138 and its engineered variants in human and mouse serum*. Acinetobacter baumannii* WHG40137 suspended in 10 mM HEPES, pH 7.4, with **(A)** 40% human serum, **(B)** 80% human serum and **(C)** mouse serum, was treated with 100 μg/mL of each lysin. After 1 h incubation at 37 °C, bacterial survival was assessed by CFU counting. The red dashed line indicates the detection limit. Data are presented as mean ± SD of three technical replicates from one representative experiment. Only statistically significant differences between treated groups and the untreated control are indicated by asterisks. Statistical analysis was performed using the Kruskal–Wallis test followed by Dunn’s multiple comparisons test based on the three technical replicates. *p < 0.05.

Overall, the engineered variants Syn138, Pad138, and CecA138 retained enhanced bactericidal activity under physiological conditions compared with the parental lysin. However, differences in activity were observed among the variants depending on the serum type and concentration tested.

### 3.4 Effect of the differences in the charge and structures of fused peptides on the differential bactericidal activity of Lyspd149

To determine whether the correlation between fused α-helical structure positively charged peptide and enhanced antibacterial activity observed for the engineered variants of LysPd138 could be extended to other lysin types, we selected two peptides from the seven peptides, namely, CecA, which forms α-helical structures and is enriched in positively charged residues, and Buforin-2 (8–21), which has a loop-like structure and contain less positively charged residues. These peptides were fused with LysPd149 at the N-terminus, resulting in CecA149 and Buf149 (Figure 4A). Structure prediction showed that CecA149 contains an α-helical fused peptide, whereas Buforin-2 (8–21) formed a partial α-helical structure upon fusion with LysPd149 (Figure 4B). Notably, the pLDDT confidence scores for the fused peptide region in Buf149 are near 70 (Supplementary Figure S4), indicating that the corresponding atomic coordinates are reliable. PSIPRED secondary structure prediction revealed that CecA has high α-helical content, whereas Buforin-2 (8–21) was predicted to have little helical content (Supplementary Figure S5).

**FIGURE 4:**
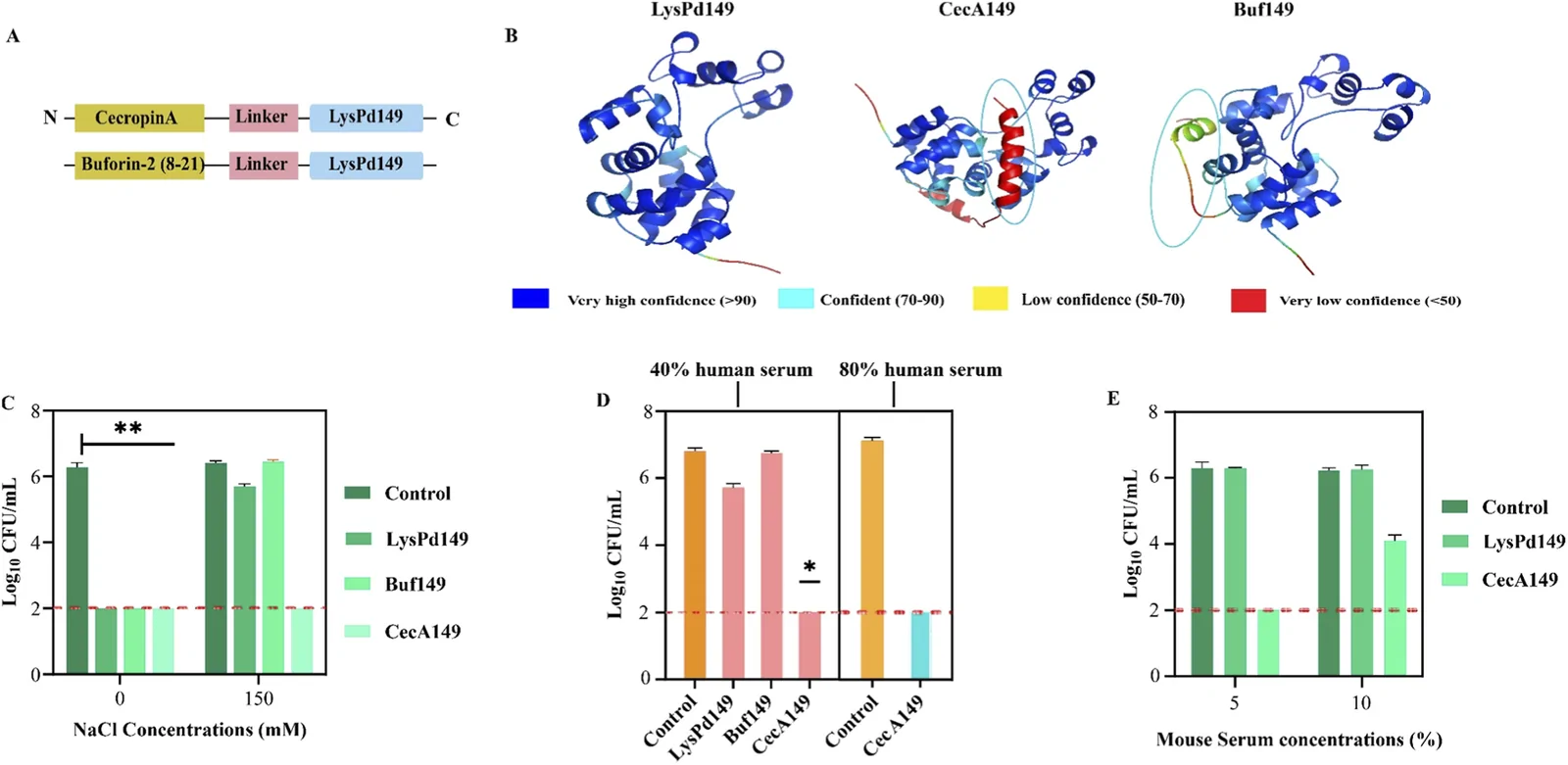
Predicted structures and Bactericidal activity of LysPd149 and its engineered variants. **(A)** Schematic diagrams of designed engineered variants. **(B)** AlphaFold-based predicted structures. The region enclosed in the blue circle indicates the fused peptide. **(C)** *Acinetobacter baumannii* WHG40137 suspended in 10 mM HEPES, pH 7.4, with and without NaCl **(D)**, suspended in human serum **(E)**, and suspended in mouse serum, was treated with 100 μg/mL of each lysin. After 1 h incubation at 37 °C, bacterial survival was assessed by CFU counting. The red dashed line indicates the detection limit. Data are presented as mean ± SD of three technical replicates from one representative experiment. Only statistically significant differences between treated groups and the untreated control are indicated by asterisks. Statistical analysis was performed using the Kruskal–Wallis test followed by Dunn’s multiple comparisons test based on the three technical replicates. *p < 0.05, **p < 0.01.

Initially, antibacterial activity was tested in 10 mM HEPES buffer at pH 7.4, with or without 150 mM NaCl (Figure 4C). In the absence of NaCl, all lysins reduced *Acinetobacter baumannii* WHG40137 to below detectable levels. In the presence of 150 mM NaCl, CecA149 eradicated the bacteria to below the detection limit, whereas LysPd149 and Buf149 significantly lost their bactericidal effects.

Next, the bactericidal effect of LysPd149, CecA149, and Buf149 was evaluated in 40% human serum. CecA149 eliminated bacteria to below the detection limit, whereas LysPd149 and Buf149 showed significant loss of activity even at 40% serum concentration. Subsequently, increasing the human serum concentration to 80% did not affect the bactericidal activity of CecA149, which continued to eliminate bacteria below the detection limit (Figure 4D). Additionally, the bactericidal activity of LysPd149 and CecA149 was evaluated in different concentrations of mouse serum against *A. baumannii* WHG40137. Results revealed that CecA149 could eliminate *A. baumannii* WHG40137 to below the detection limit at 5% serum; however, 10% serum significantly inhibited the bactericidal activity of both lysins (Figure 4E).

Overall, CecA149 exhibited greater bactericidal activity under physiological conditions such as high NaCl concentration and human serum.

### 3.5 Stability of CecA149 in human plasma over time

To assess whether the engineered variants offer enhanced activity compared with the sole peptide under physiological conditions, we selected CecA149 as a representative engineered variant and compared its activity with the CecA peptide after incubation in human plasma. CecA149, at a final concentration of 3.8 µM (equivalent to 100 μg/mL), retained strong antibacterial activity after incubation in human plasma, whereas CecA at the same molar concentration exhibited a significant reduction in antibacterial activity. After 2 h of incubation, CecA149 resulted in a 3.4-log reduction in bacterial counts compared with the HEPES-treated group, whereas CecA showed only a 1.1-log reduction. After 3 h, CecA149 maintained a 2.5-log reduction, while CecA exhibited only a 0.6-log reduction. At 5 h, the antibacterial activity of both antimicrobials was substantially reduced, with CecA149 and CecA showing 0.3-log and 0.1-log reductions, respectively (Figure 5). These results demonstrate that CecA149 retained higher antibacterial activity than CecA throughout the incubation period in human plasma. At 5 h, both antimicrobials lost significant activity.

**FIGURE 5:**
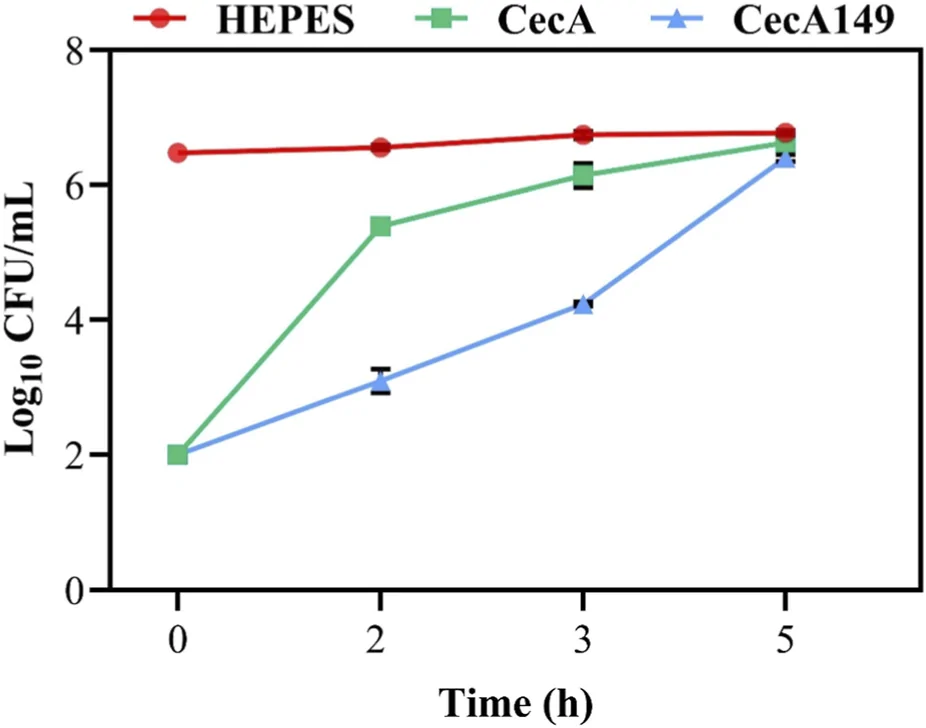
Residual activity of CecA149 and CecA after incubation in 50% human plasma for 0, 2, 3, and 5 h at 37 °C. After incubation, proteins were mixed with *Acinetobacter baumannii* WHG40137 and incubated for 1 h at 37 °C. Bacterial survival was assessed by CFU counting. Data are presented as mean ± SD of three technical replicates from one representative experiment.

### 3.6 Active engineered variants increase the OM permeability of *Acinetobacter baumannii* WHG40137

In order to find a possible mechanism behind the enhanced activity in 150 mM NaCl, a widely used membrane permeability assay, NPN uptake (Li Q. et al., 2021; Lim et al., 2024), was used to test whether the fused peptides aide permeabilization of the bacterial membrane. NPN uptake level in *Acinetobacter baumannii* WHG40137 suspended in 10 mM HEPES-NaOH (pH 7.4) was measured in the presence or absence of 150 mM NaCl (Supplementary Figure S6). In the absence of NaCl, increased NPN uptake was observed in *A. baumannii* WHG40137 treated with 100 μg/mL LysPd138, LysPd149 or their engineered variants (Supplementary Figure S6A). However, in the presence of NaCl, only Syn138, Pad138, CecA138, and CecA149 showed significantly higher NPN uptake, achieving 2.6-, 2.3-, 2.8-, and 1-fold increases, respectively (Supplementary Figure S6B). These findings suggest that engineered variants contain α-helical fused peptide retain high outer membrane permeability in high-salt environment.

## 4 Discussion

As alternative antimicrobial agents, bacteriophage-derived lysins have attracted a lot of attention. However, their therapeutic application against Gram-negative bacteria is hindered by losing bactericidal activity in the presence of physiological fluids, such as high NaCl concentrations and sera (Yao et al., 2025; Chu et al., 2022). Recent studies have increasingly focused on protein engineering and peptide fusion strategies to enhance the bactericidal activity of lysins against Gram-negative pathogens (Yao et al., 2025; Kim J. et al., 2025; Lim et al., 2024; Vasina et al., 2024; Hong et al., 2025; Gerstmans et al., 2023; Sui et al., 2023). To date, over 15,700 AMPs have been identified with antimicrobial activity (Pirtskhalava et al., 2021). Screening such a vast database to find the optimal combination of AMPs and lysins would be a lengthy and challenging task. Consequently, new construction and screening platforms are essential to accelerate lysin development and screening, addressing the urgent need due to the rapid rise of multidrug-resistant bacteria (Sui et al., 2023). Therefore, a deeper understanding of the contributing factors of antimicrobial peptides is required for the rational choice of suitable peptides to engineer the lysins.

In this study, a short flexible (AG)_3_ linker was selected to fuse the peptides with the lysin, as we reasoned that Ala-Gly-based linkers can provide sufficient flexibility while minimizing steric interference between fused domains. Similar linkers have been successfully used in previous peptide-lysin fusion studies, and enhanced lysin activity after peptide fusion has been reported (Abdelkader et al., 2020; Gerstmans et al., 2020). Using this fusion strategy, analysis of seven engineered LysPd138 variants suggests that differences in antibacterial activity are associated with differences in the predicted structural characteristics and net positive charge of the fused peptides. Computational structure prediction using AlphaFold2 and PSIPRED suggested that the variants (Syn138, Pad138, and CecA138) exhibiting enhanced activity in 150 mM NaCl solution and sera were fused with peptides predicted to contain predominantly α-helical structures (Figure 2E). This observation was further examined by fusing two representative peptides with another lysin, LysPd149 (Figure 4). Notably, Buf149 contained a peptide predicted to adopt a partial α-helical structure but possessed relatively few positively charged residues and did not retain bactericidal activity under physiological conditions. Together, these observations suggest that the predicted α-helical propensity and positive charge of the fused peptide are associated with improved antibacterial activity of engineered lysins under physiological conditions. However, it is also possible that other physicochemical characteristics of the fused peptides, such as amphipathicity, contribute to the observed activity (Bui Thi Phuong et al., 2024). In addition, Buforin-2 (8–21) was predicted to adopt a loop-like structure when fused to LysPd138 but a partially α-helical structure when fused to LysPd149, suggesting that peptide conformation may depend on the lysin type. Based on these observations, it may be possible to develop a strategy for lysin engineering using structure prediction and charge analysis, as illustrated in Figure 6. Before the expression and purification of peptide-lysin fusion proteins, computational structure prediction and charge analysis could be used to select peptide candidates with a higher likelihood of improving lysin activity under physiological conditions. Although further validation using additional peptide-lysin combinations is required, this workflow provides a proof-of-concept that computational analysis may improve the efficiency of lysin engineering and reduce the time and cost associated with conventional trial-and-error approaches.

**FIGURE 6:**
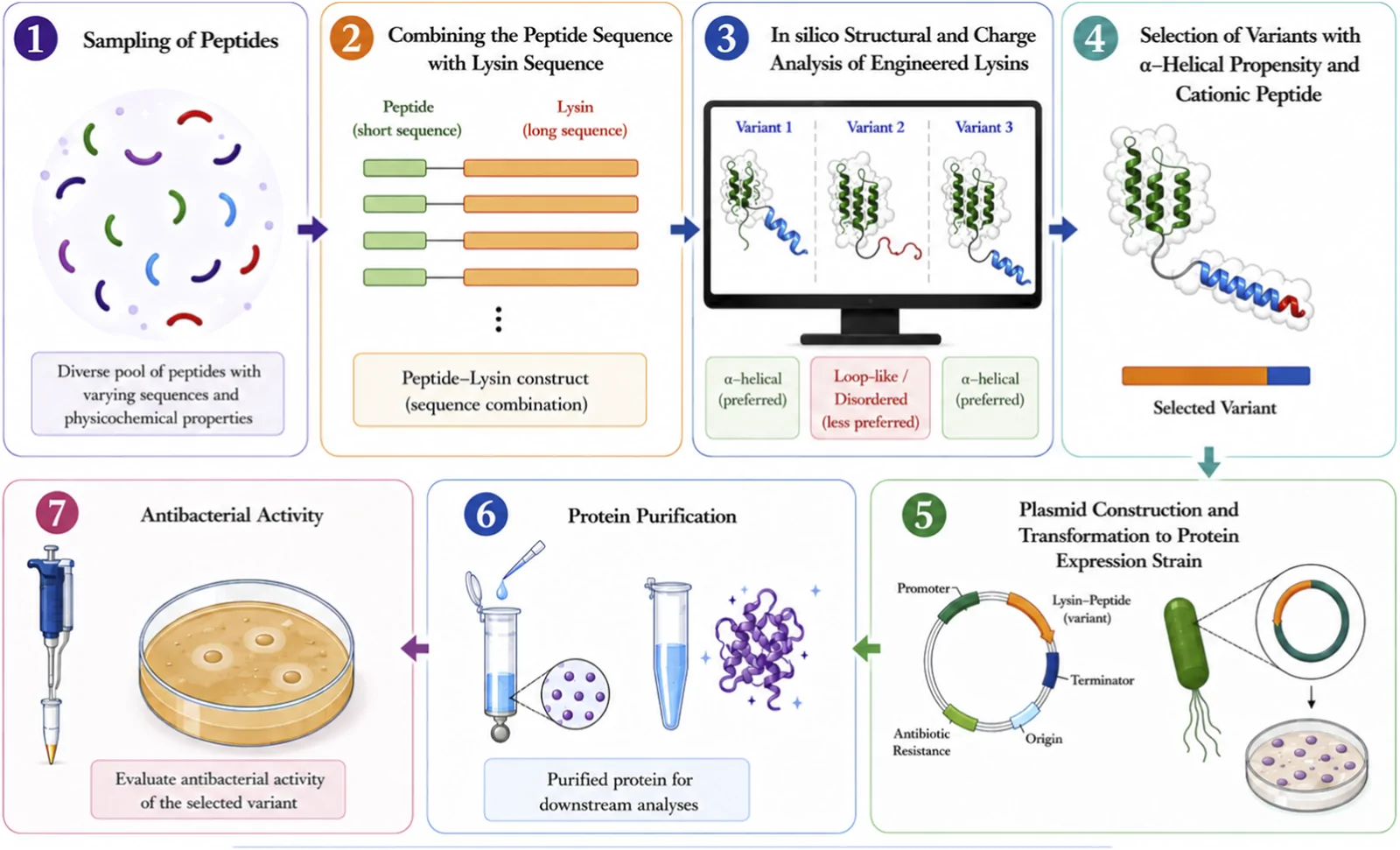
Schematic representation of the proposed proof-of-concept platform for lysin engineering, highlighting the rational selection of an engineered variant active in physiological fluids against Gram-negative bacteria, particularly *Acinetobacter baumannii*.

Our results suggest that AI-based structure prediction tools, such as AlphaFold2, may provide useful guidance for lysin engineering. In this study, the structures of the peptide-lysin fusion proteins were first predicted using AlphaFold2. Notably, the fused peptide regions generally exhibited low pLDDT scores (<50), indicating considerable uncertainty in the predicted local structures. Therefore, the AlphaFold2 models were not interpreted as experimentally validated structures. Instead, the predicted secondary structures were independently assessed using PSIPRED, which showed a similar trend in the predicted α-helical or loop-like conformations of the fused peptides. The agreement between these two computational approaches, together with the observed antibacterial activities of the engineered lysins, suggests that careful analysis of AlphaFold2-based predictions combined with complementary computational methods and experimental evaluation may provide useful guidance for the selection of peptide candidates during lysin engineering.

An α-helical structure can enhance antimicrobial activity by forming distinct positively charged, hydrophilic, and hydrophobic regions on its surface, which may promote interaction with the target cell membrane (Quagliata et al., 2025). Consistent with this property, the NPN uptake assay (Supplementary Figure S6 **)** showed that, in the absence of NaCl (10 mM HEPES), both the native lysin and all engineered variants exhibited high outer membrane permeabilization activity. This observation is consistent with their similarly high antibacterial activities under low-tonicity conditions (Supplementary Figure S1), suggesting that the bacterial outer membrane is more susceptible to lysin-mediated disruption in the absence of physiological salt concentrations. In contrast, in the presence of 150 mM NaCl, only the engineered lysins fused with peptides predicted to possess greater α-helical propensity and enriched in positively charged residues retained comparatively higher outer membrane permeabilization activity, whereas the native lysin and variants containing loop-like peptides showed reduced permeabilization. These observations suggest that the fused peptides may help maintain outer membrane-disrupting activity under physiological salt conditions, which may be associated with the improved antibacterial activity of the corresponding engineered lysins. Similar associations between improved outer membrane-disrupting properties and enhanced antibacterial activity have been reported for other engineered lysins (Gerstmans et al., 2023; Garcia Torres et al., 2025). Furthermore, previous studies have demonstrated that colistin-mediated outer membrane permeabilization can potentiate lysin activity (Zhang et al., 2024). Collectively, these findings support the hypothesis that overcoming the outer membrane barrier is likely one of the important factors contributing to lysin activity under physiological conditions.

Another interesting observation was that mouse serum appeared to inhibit the activity of both native and engineered lysins more strongly than human serum (Figures 3C, 4E). This finding suggests that mouse serum may contain components that interfere with lysin activity to a greater extent than human serum. One possible explanation is the presence of proteolytic enzymes or other inhibitory factors that partially reduce the activity of the engineered lysins, although this remains hypothetical. Interestingly, CecA149 retained higher antibacterial activity than the CecA peptide alone after incubation in human plasma, suggesting that fusion of the peptide to the lysin may provide advantages over the peptide by itself under physiological conditions. The mechanism underlying this observation remains unclear and may involve multiple factors, including differences in protein stability, interactions with serum components, or interactions with bacterial membranes.

In this study, the structural analyses were based primarily on computational predictions generated by AlphaFold2 and PSIPRED. As the fused peptide regions generally exhibited low pLDDT scores and the predicted secondary structures were not experimentally verified, additional experimental techniques, such as circular dichroism spectroscopy and helix-disrupting mutations, would provide valuable experimental validation of the predicted peptide conformations. Similarly, although the observed association between predicted α-helical propensity, positive charge, and antibacterial activity was reproduced using two lysin types, the present results demonstrate correlation rather than causation, and the proposed mechanism of enhanced membrane interaction and permeabilization is supported primarily by the NPN uptake assay. Future studies will be required to experimentally determine the relative contributions of α-helicity, charge distribution, amphipathicity, conformational flexibility, and other physicochemical properties, as well as to validate the proposed mechanism using complementary membrane interaction and permeabilization assays. Furthermore, understanding the factors responsible for mouse serum-mediated inhibition will be important for further optimizing engineered lysins, particularly because their therapeutic efficacy is typically evaluated in mouse infection models. Finally, although the proposed engineering strategy was evaluated using two lysins, the antibacterial activity was assessed primarily using a single *A. baumannii* strain. Therefore, the broader applicability of this computational peptide-selection strategy should be validated using additional lysins, a wider collection of multidrug-resistant clinical isolates, and other Gram-negative bacterial species.

## 5 Conclusion

In conclusion, this study explored the potential of protein structure prediction and charge analysis to identify peptide characteristics associated with the activity of engineered lysins against *A. baumannii* under physiological conditions. The results suggest that peptides predicted to exhibit greater α-helical propensity and enriched in positively charged residues were associated with improved antibacterial activity when fused to two different lysins. These findings provide a proof-of-concept that integrating computational structure prediction with charge analysis may facilitate the rational selection of peptide candidates to engineer lysins against Gram-negative bacteria, particularly *A. baumannii*, to improve their activity in physiological fluids.

## Data Availability

The original contributions presented in this study are included in the article/Supplementary Material. Further inquiries can be directed to the corresponding author.
