# Supplementary material for: Experimental screening and structure-informed engineering of peptide-fused bacteriophage lysins with enhanced activity against Gram-negative bacteria
Source: Front Pharmacol. 2026 Sep 21;17:1918219. doi: 10.3389/fphar.2026.1918219 (PMC13634823; doi:10.3389/fphar.2026.1918219)
Supplement: Supplementary file 1 [file Supplementaryfile1.docx]

**Supplementary data**

**Experimental Screening and Structure-Informed Engineering of Peptide-Fused Bacteriophage Lysins with Enhanced Activity against Gram-Negative Bacteria**

Ali Murtaza ^a,b#^, Fangfang Yao ^c#^, Aqib Saeed ^a,b^, Xiaohong Li ^a^, Nelson Odiwuor ^a^, Xinjing Du ^a^, Changchang Li ^a^, Junhua Li ^a^, and Hongping Wei ^a,b*^

^a^ WHP Innovation Lab, State Key Laboratory of Virology and Biosafety, Wuhan Institute of Virology, Chinese Academy of Sciences, Wuhan, Hubei 430207, China

^b^ University of Chinese Academy of Sciences, Beijing, 100049, China

^c^ Stomatological Hospital of Xiamen Medical College, Xiamen Key Laboratory of Stomatological Disease Diagnosis and Treatment, Xiamen, China.

For correspondence: *Hongping Wei: E-mail: hpwei@wh.iov.cn;

^#^These authors contributed equally to this work

| **peptide** | **Sequence** | **Predicted net charge at pH 7** |
| --- | --- | --- |
| G8 | GWKRKRFG | +4 |
| DP7 | VQWRIRVAVIRK | +4 |
| SynSaf-P56 | AGKEKIRKKLKNEIKKKWRKAVIAW | +9 |
| E7K_I9K_IsCT | ILGKIWKGKKSLF | +4 |
| PaDBS1R6 | PMARNKKLLKKLRLKIAFK | +8 |
| CecropinA | KWKLFKKIEKVGQNIRDGIIKAGPAVAVVGQATQIAK | +6 |
| Buforin-2 (8-21) | LQFPVGRVHRLLRK | +4.1 |

**Table S1**: predicted net charge of the selected peptides

**Figure S1**: (**A, B)** Analysis of purified proteins on 12% SDS PAGE. The M lane indicates the marker (**C**) Concentrations measured with NanoDrop or BCA. (**D**) Antibacterial activity of LysPd138 and its engineered variants in 10 mM HEPES buffer at pH 7.4 without 150 mM NaCl. the red line indicates the detection limit.

**Figure S2**: pLDDT confidence plot of engineered variants of LysPd138. The structure presented in S2 is rank 1.

**Figure S3**: PSIPRED-based secondary structure prediction of engineered variants of LysPd138. The residues in the dashed box indicate the fused peptide.

**Figure S4**: pLDDT confidence plot of engineered variants of LysPd149. The structure presented in S2 for LysPd149, Buf149 and CecA149 is rank 1.

**Figure S5**: PSIPRED-based secondary structure prediction of engineered variants of LysPd149. The residues in the dashed box indicate the fused peptide.

**Figure S6**. Membrane-penetrating activity of the 100 µg/mL LysPd138, LysPd149 and their variants against A. baumannii WHG40137 suspended in **(A**) HEPES-NaOH, **(B)**HEPES-NaOH, 150 mM NaCl. All experiments were performed in triplicates. Data are expressed as mean ± SD.

**Parameters for protein structure prediction**

colabfold_batch \--model-type auto \--num-recycle 3 \--recycle-early-stop-tolerance 0.5 \--msa-mode MMseqs2 \--pair-mode unpaired+paired \--num-models 5 \--rank plddt \input.fasta \output_directory

**In Vitro antibacterial assays under different conditions.**

For the experiment in NaCl and 40% human serum, the protein was diluted to 333 µg/mL. Similarly, the IM NaCl solution was diluted to 750 mM in HEPES buffer, ensuring the HEPES concentration remained 10 mM in the solution. The preparation of the bacterial cells is described in the main manuscript text. The following volumes were added from the prepared solutions to the reaction solution (200 µL). Notably, the bacteria were first combined with the NaCl, human serum or mouse serum and then the protein (lysin) was added to the reaction solution.

**Reaction solution for the killing assay in NaCl**

|  | Lysin (µL) | Prepared Bacteria (µL) | HEPES buffer (µL) | NaCl (µL) |
| --- | --- | --- | --- | --- |
| Treated group (0 mM NaCl) | 60 | 100 | 40 | - |
| Control (0 mM NaCl) | - | 100 | 100 | - |
| Treated group (150 mM NaCl) | 60 | 100 | - | 40 |
| Control (150 mM NaCl) | - | 100 | 60 | 40 |

**Reaction solution for the killing assay at different concentrations of lysin in the presence of 150 mM NaCl**

|  | Lysin (µL) | Prepared Bacteria (µL) | HEPES buffer (µL) | NaCl (µL) |
| --- | --- | --- | --- | --- |
| Treated group (100 µg/mL) | 60 | 100 | - | 40 |
| Treated group (85 µg/mL) | 51 | 100 | 9 | 40 |
| Treated group (70 µg/mL) | 42 | 100 | 18 | 40 |
| Treated group (50 µg/mL) | 30 | 100 | 30 | 40 |
| Control | - | 100 | 60 | 40 |

**Reaction solution for the killing assay in 40% human serum**

|  | Lysin (µL) | Prepared Bacteria (µL) | HEPES buffer (µL) |
| --- | --- | --- | --- |
| Treated group | 60 | 82 | 58 |
| Control | - | 82 | 118 |
|  |  |  |  |

Similarly, for experiments in 80% human serum or mouse serum, the protein was diluted to 526 µg/mL. The preparation of the bacterial cells is described in the main manuscript text. The volumes given in the following tables were combined to make a total of 200 µL reaction solution.

**Reaction solution for the killing assay in 80% human serum**

|  | Lysin (µL) | Prepared Bacteria (µL) | HEPES buffer (µL) |
| --- | --- | --- | --- |
| Treated group | 38 | 162 | - |
| Control | - | 162 | 38 |

**Reaction solution for the killing assay in mouse serum**

|  | Lysin (µL) | Prepared Bacteria (µL) | HEPES buffer | Mouse serum (µL) |
| --- | --- | --- | --- | --- |
| Treated group 5% | 38 | 100 | 52 | 10 |
| Treated group 10% | 38 | 100 | 42 | 20 |
| Control 5% | - | 100 | 90 | 10 |
| Control 10% | - | 100 | 80 | 20 |

**5× Tris-HCl Solution**

Dissolved 12.114 g Tris-base and 146.1 g NaCl in 900 mL of sterile water. Adjusted pH to 8.0 with concentrated HCl, then brought to a final volume of 1 L. Filter sterilized using a 0.22 µM filter and stored at room temperature.

**Equilibration/Binding Buffer**

20 mM Tris-HCl, 0.5 M NaCl, 20 mM imidazole, pH 8.0 (Prepare by mixing 200 mL of 5× Tris-HCl and 20 mL of 1 M imidazole, then fill up to 1 L with ultrapure water).

**IM HEPES buffer**

To make 1M HEPES buffer, 23.8 gram HEPES was dissolved in 100 mL of distilled water. The pH was adjusted to 7.4 by adding sodium hydroxide. The buffer was filter-sterilized.

**Protein dialysis buffer**

IM HEPES buffer was diluted 100 times in distilled water to make a 10 mM HEPES buffer for protein dialysis.
